# Bleaching protection and axial sectioning in fluorescence nanoscopy through two-photon activation at 515 nm

**DOI:** 10.1038/s41467-024-51160-9

**Published:** 2024-08-29

**Authors:** Jan-Erik Bredfeldt, Joanna Oracz, Kamila A. Kiszka, Thea Moosmayer, Michael Weber, Steffen J. Sahl, Stefan W. Hell

**Affiliations:** 1 Department of NanoBiophotonics, Max Planck Institute for Multidisciplinary Sciences, Göttingen, Germany; 2 Georg-August University School of Science (GAUSS), University of Göttingen, Göttingen, Germany; 3 Department of Optical Nanoscopy, Max Planck Institute for Medical Research, Heidelberg, Germany

**Keywords:** Super-resolution microscopy, Biological fluorescence

## Abstract

Activation of caged fluorophores in microscopy has mostly relied on the absorption of a single ultraviolet (UV) photon of ≲400 nm wavelength or on the simultaneous absorption of two near-infrared (NIR) photons >700 nm. Here, we show that two green photons (515 nm) can substitute for a single photon (~260 nm) to activate popular silicon-rhodamine (Si-R) dyes. Activation in the green range eliminates the chromatic aberrations that plague activation by UV or NIR light. Thus, in confocal fluorescence microscopy, the activation focal volume can be matched with that of confocal detection. Besides, detrimental losses of UV and NIR light in the optical system are avoided. We apply two-photon activation (2PA) of three Si-R dyes in different superresolution approaches. STED microscopy of thick samples is improved through optical sectioning and photobleaching reduced by confining active fluorophores to a thin layer. 2PA of individualized fluorophores enables MINSTED nanoscopy with nanometer-resolution.

## Abstract (web summary)

Activation of caged fluorophores has mostly relied on the absorption of either a single ultraviolet photon or two NIR photons. Here, the authors show that two green photons (515 nm) can substitute for a single photon ( ~ 260 nm) to activate rhodamine-based dyes, thus enabling STED imaging in thick samples.

## Introduction

**Fig. 1:**
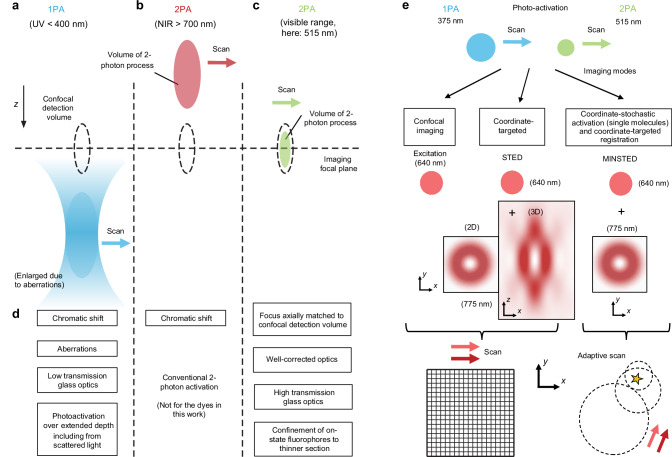
Rationale for a preferred two-photon fluorophore photoactivation in the visible wavelength range, and nanoscopic imaging modalities. Optical sectioning is achieved by the confocal detection principle (use of a pinhole), in addition to selective activation of photoactivatable fluorophores in targeted sample volumes. Only activation at the visible (green) wavelength allows matching of the rendered fluorescence to the detection volume in *z* (dashed lines), because the focusing performance is highly corrected for visible light and especially in the green range. Ultraviolet (UV) and near-infrared (NIR) light is focused with substantial chromatic offsets. **a** One-photon activation (1PA) with UV light leads to continuous activation along the beam path. **b** For 2PA of fluorophores, shown here for the case of near-infrared light, the activated volume is sharply confined as the volume with sufficiently high photon density for ensuing the 2-photon activation (2PA) process. This leads to background suppression outside. **c** 2PA with visible light, the concept explored in this work for sectioning in fluorescence nanoscopy with 515-nm femtosecond pulses. **d** Features (advantages and disadvantages) of the visible-light 2PA vs. UV 1PA approach. **e** Examples of imaging modes demonstrated in conjunction with 515-nm 2PA. STED (stimulated emission depletion), MINSTED: nanoscopy concepts.

Effective background suppression is a key requirement in many advanced optical imaging applications^1^. Especially when three-dimensional (3D) imaging of larger sample volumes is required, axial sectioning with a confocal pinhole^2^ continues to be the method of choice in both regular scanning^3^ and superresolution^4^ fluorescence microscopy. Confocal detection greatly confines fluorescence detection to a nearly diffraction-limited focal volume around the lens focus. To achieve additional selectivity in the axial direction, it is highly attractive to use photoactivatable fluorophores since they allow confining the generation of the fluorescence signal to selected focal volumes or layers. In order to operate well, photoactivation should therefore axially match the confocal detection volume as much as possible (compare Fig. 1a–c).

In this respect, widely used approaches to activation have been rather deficient. Both for one-photon activation (1PA), typically at ultraviolet (UV) wavelengths^5–8^, and two-photon activation (2PA) with light in the near-infrared (NIR) spectral region^9–11^, chromatic longitudinal aberrations lead to poor overlap of the activated sample volume with the one from where fluorescence is detected (Fig. 1a, b). Attempts to remedy the axial mismatch by deliberate defocusing are unsatisfactory, because the focal spot sizes become unduly large, which also leads to unnecessarily large activated volumes, and exacerbates bleaching^12^. Therefore, it is highly advantageous to activate with a wavelength for which the lens is corrected, together with the fluorescence excitation and emission wavelength.

In scanning STED (stimulated emission depletion) microscopy^13–15^ and related methods based on the RESOLFT principle^16^, the imaging resolution is achieved by creating an “on”-“off” fluorophore state contrast at sub-diffraction scales^17^ with an additional light pattern (frequently donut-shaped), which inhibits fluorescence in the outer parts of the focal excitation spot and exclusively allows the occupation of the fluorescent “on” state for those fluorophores positioned at the center of the light pattern. Outside the coordinate that is targeted at the respective scan step, virtually permanent occupation of the dark “off” state is ensured by the STED light. Following signal collection, scanning then shifts the targeted coordinate and defines the contrast for a new constellation of on- and off-fluorophores, followed by a new coordinate and on-off constellation, and so on. In standard STED microscopy, the “on” state is the state from which a fluorescence photon is emitted, whereas the “off” state is the ground state of the fluorophore.

The concept of transferring fluorophores with light to distinct states is instructive also when thinking how best to realize and optimize superresolving approaches like STED in practical respects. One important factor that poses challenges is photobleaching, i.e., the irreversible loss of fluorophores from the imaging process. Imperfect photophysical behavior, as far as the conceptual strictly two-state switching outlined above is concerned, eliminates fluorophores with progressive duration of illumination. The effect is increasingly prominent at high intensities, and photobleaching ultimately limits resolution performance in standard STED imaging^18^. Previously, the MOST (multiple off-state transitions) nanoscopy concept^19^, using transitions of photoswitchable fluorescent proteins, “shelved” fluorophores in a more light-inert “protected” state as a remedy. Thereby, the excitation light and the resolution-providing STED light pattern exert their state-cycling action only on the relevant, active fluorophores near the center of the scanned pattern. More generally, transferring only those fluorophores which are part of the “resolution action” to a state in which they can be part of the on-off cycling, is beneficial as it minimizes the impact of bleaching and enhances image contrast. This becomes especially apparent when considering a sample with a non-negligible axial (*z*) extent. In this situation, it is unavoidable in practice that secondary signal contributions arise from sample regions above and below the current scan step’s coordinate which are not entirely rejected by the confocal detection pinhole.

Selective activation of the momentarily addressed sample layer (Fig. 1) provides a cleaner situation to image, without the signals from resolved fluorophores being affected by the out-of-plane background from fluorophores in not yet targeted layers. Photoactivatable fluorophores undergo a structural change upon absorption of light and become able to emit fluorescence^5–8^. The wavelength required for activation typically lies in the blue to UV spectral range, utilising light at ≲400 nm wavelengths (Fig. 1a). The application of these short wavelengths is challenged, however, by pronounced optical aberrations and poor transmission of the lens glass and glue employed in microscopy optical elements. In addition, providing the activation energy through a one-photon absorption process does not strongly select the focal layer only, as activation occurs along the entire beam path, above and below focus, and with contributions also from scattered light (Fig. 1a).

2PA of photoactivatable fluorescent dyes, used as a means of selectively addressing individual layers, has the potential to deliver improved contrast (signal-to-background) due to the restriction of the activated volume. The application of 2PA has been largely limited to the uncaging of chemicals^20,21^ and the field of optogenetics^22,23^. Less work has focused on the photoactivation of dyes or their photo-conversion, and previous work discussing the 2PA of coumarin fluorophores^9^, of photoactivatable GFP^10^, or the conversion of fluorescent cyanine-based dyes^11^, limited itself to the customary two-photon wavelength regime > 700 nm (Fig. 1b). We chose not to operate at the classical NIR wavelengths (Fig. 1b) however, but to carry out the activation with 515 nm femtosecond pulses (Fig. 1c), moving to the visible regime for appropriate fluorophores. Indeed, 2PA of negatively-switching reversibly photoswitchable fluorescent proteins (RSFPs) at yellow wavelengths and its use for confocal imaging were recently described^24^. We reasoned that the required activation energy typically provided by UV photons could be delivered by green photons in a multiphoton process. The application of green light for 2PA in superresolution microscopy has not been described to our knowledge. As we show, the two-photon nature of the activation process, when implemented with green light, comes with a number of advantages (Fig. 1d), including confinement of the activated fluorescent-capable state to thinner sections. Demonstrated here for silicon-rhodamine (Si-R) dyes^5–8^ which can be employed with various labeling approaches, our strategy holds considerable promise for future extensions of the highly resolving fluorescence nanoscopy tools to deeper layers when combined with other important developments such as adaptive-optics focusing^25,26^.

Here, we demonstrate various superresolution modalities (Fig. 1e) in conjunction with 2PA effected by green (515 nm) light, thereby creating an axially matched situation of activation and confocal detection volume (Fig. 1c, d). This enables STED imaging^13–15^ at improved contrast in cellular and tissue samples. The approach also benefits from a substantial photobleaching reduction compared to regular 1PA at 375 nm, especially when scanning 3D volumes. Only the fluorophores in the 2PA-targeted confined section that is relevant for the imaging at any given time are switched to “active”, with the others remaining largely inert to the excitation and STED light. The presented strategy, utilizing a widely used class of photoactivatable (PA) Si-R dyes^5–8^, is also compatible with latest superresolution developments such as nanometer-resolution MINSTED^27^.

## Results

### Properties of photoactivatable silicon rhodamine dyes, and technical implementation considerations with microscope optics

PA Si-R dyes^5^ feature high-contrast activation, high fluorescent quantum yield, and low photobleaching. These fluorophores usually exhibit absorption maxima around 640 nm followed by fluorescence emission at 660–690 nm upon activation by UV or blue light (370–405 nm). Initially, the PA Si-R dyes were developed for single-molecule localization nanoscopy and later modified for coordinate-targeted schemes, notably STED and MINSTED^27^. We investigated two types of PA Si-R dyes, caged PA Si-R derivatives (ONB-2SiR^7^, HCage 620^8^), for which caging groups are cleaved upon illumination, and a PA Si-R derivative which is activated via a protonation after exposure to the activation light (pPA-SiR^6^).

As already discussed, the use of deep-blue and UV light is problematic from an implementation perspective as common microscope objective lenses are not designed for such short wavelengths (compare Supplementary Fig. 1). The longitudinal color error of even well-corrected objective lenses can be substantial outside the classical design range in the visible part of the spectrum. In addition to a considerable shift of the focus position along the optical axis for the UV light, the activation beam cannot be tightly focused due to aberrations. Poor control of the activation volume leads to significant out-of-focus fluorescence and therefore reduced contrast of the acquired images, as a result of higher sample-dependent background. Moreover, the transmission through glass and lens cement is low and there is an increased risk of damage to optical elements such as optical fibers or cemented doublet lenses. For coordinate-targeted schemes such as STED or MINSTED which typically have employed de-activation light in the far-red, NIR spectral range (e.g., 775–800 nm), the chromatic shifts that manifest in the opposite direction for red and far-red wavelengths cause additional technical problems with achieving focal overlap of the beams in the axial dimension (*z*). In STED microscopy, for example, the excitation beam is intentionally defocused to match the axial position of the 775 nm beam. Despite this defocusing, the final STED image is not affected due to the STED beam’s ability to prevent fluorescence from out-of-focus regions reaching the detector. Confocal detection may also contribute to image quality, but for high-resolution images, it is the STED beam that dictates the targeted region of on-state fluorophores.

**Fig. 2:**
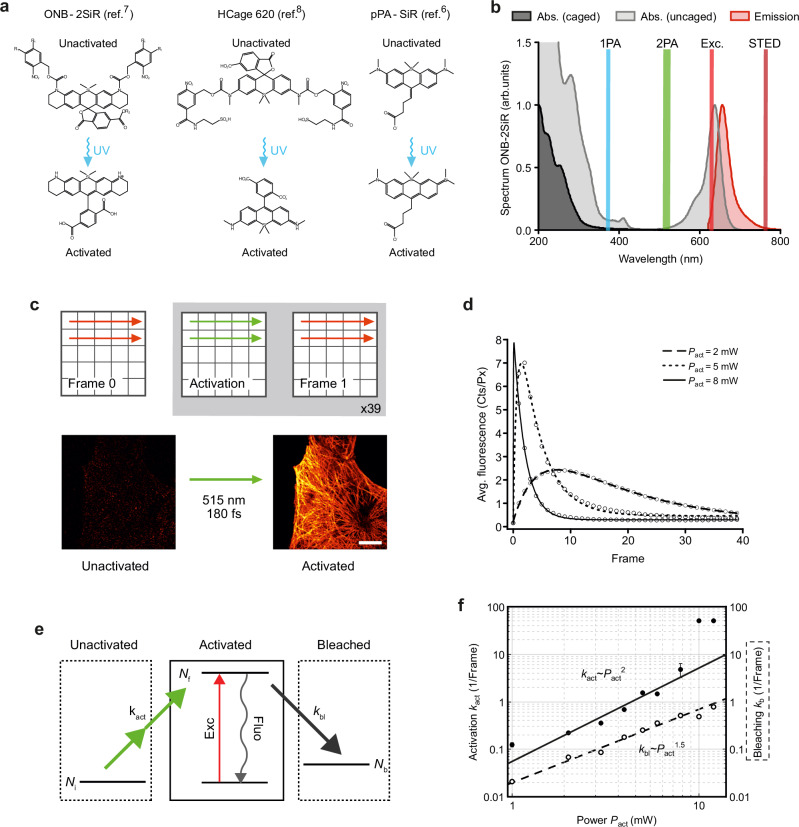
The silicon rhodamine dyes ONB-2SiR, HCage 620 and pPA-SiR can be efficiently photoactivated by a two-photon process with 515 nm light. **a** Chemical structures of ONB-2SiR, HCage 620 and pPA-SiR before (upper row) and after activation (lower row). **b** Absorption spectrum of the unactivated caged form (dark gray), and absorption and emission spectra of the activated uncaged form (light gray, red) for ONB-2SiR. The green and blue lines centered at 515 nm and 375 nm indicate the laser light employed for the 2PA and 1PA, respectively. Exc., STED: excitation and STED wavelengths. **c** Experimental sequence: Frame 0: reference scan with the excitation beam alone to detect residual background fluorescence of unactivated dye; Activation: scan with 515-nm femtosecond laser to uncage (activate) fluorophores; Frame 1: readout of the fluorescence signal with the excitation beam. Below: the images of tubulin before and after activation (10 × 10 µm^2^, 256 × 256 pxl^2^). Scale bar: 10 µm. All beams were diffraction-limited, the activation and readout sequence were repeated 39 times. **d** Average fluorescence signal vs. number of consecutive readout frames after activation scans for different activation powers *P*_act_. For low activation power, the signal initially increases. Later, photobleaching is dominant, resulting in a signal decrease. **e** A 3-level system, which takes the activation and bleaching process into account, is sufficient to describe the observed signal behavior (curves in **d**). **f** Activation and bleaching rate extracted from fits to the data in **d** as a function of activation power. The power scaling indicates a two-photon process for activation, and more complex photobleaching behavior^18^. Error bars represent standard error of the mean (s.e.m.), which is similar to the marker size in many instances. Source data are provided as a Source Data file.

A 2PA with visible light offers a conceptually straightforward alternative to the 1PA. As the two-photon process requires high energy density due to a small nonlinear absorption cross-section and/or short intermediate state lifetime, femtosecond pulses are preferred. Upon examination of the activation and absorption spectrum of the ONB-2SiR dye (Fig. 2a, b) we reasoned that optimal 2PA should be achievable at wavelengths in the range around 480–500 nm. This wavelength satisfies the condition of a high cross-section of 2PA—assuming it corresponds to 1PA absorption at ~250 nm—and low one-photon excitation of activated compounds, which might otherwise lead instantly to bleaching of the fluorophores that had just been activated. Looking for appropriate laser sources we employed frequency doubling of an ytterbium-doped fiber oscillator with a fundamental wavelength at 1030 nm. The 515 nm femtosecond laser provides <180 fs pulses with a 20 MHz repetition rate (Halite, Fluence Technology).

### PA silicon rhodamines can be two-photon-activated with green 515 nm light

First, we investigated the activation process of ONB-2SiR (Fig. 2a) by 515 nm femtosecond pulses to assess the corresponding nonlinearity order *n* of the process on the activation light power (activation $\sim {P}^{n}$) and the optimal power value that maximizes the activated fluorescence signal. To this end, we imaged tubulin in fixed U-2 OS cells immunostained with ONB-2SiR as the dye in a dedicated experimental sequence (Fig. 2c). Note that the later imaging experiments relied on just a single activation scan at appropriate power and pixel dwell time to provide a suitable activation light dosage (Supplementary Table 1).

Using a custom-built microscope setup (Supplementary Fig. 2), we initially collected a reference confocal image to measure an inherent background fluorescence signal of the unactivated dye by scanning the sample with the excitation beam (*λ* = 640 nm, *τ* ≈ 100 ps, *P* = 8 mW, *f* = 40 MHz, *t*_dwell_ = 30 μs, “Frame 0“). Typically, the signal was negligible. Then, we activated the compound by scanning the same field of view (FOV) with activation light (*λ* = 515 nm, *τ* ≈ 200 fs, *P* = 0-8 mW, *f* = 20 MHz, *t*_dwell_ = 100 μs, “Activation”), during which the detector was switched off. In the following scan, we detected a fluorescence image by scanning the same FOV with the excitation beam (parameters as before, “Frame 1”). The sequence of activation and subsequent readout was then repeated a further 38 times for each activation power. For each activation power measured, a fresh region of the sample was used, with different cell confluency and therefore total fluorescence signal.

To enable comparisons, we calculated the average fluorescence signal per pixel for pixels above noise level. The results (Fig. 2d) reveal that the initial activations typically increased the fluorescence, as a fraction of dyes was already activated during a single scan. Upon reaching a maximum, photobleaching of already activated molecules became dominant and the signal began to decline with further exposure to the activation light. The registered dependence can be modeled by a 3-state system (Fig. 2e) which comprises the electronic states *N*_i_, *N*_f_, and *N*_b_ corresponding to an initial non-activated, a fluorescent and a bleached fluorophore, respectively. Repeated scanning with the excitation laser showed no significant changes in signal, therefore only the activation and bleaching by the activation laser were taken into account. Under the assumption that the detected signal is proportional to the number of activated fluorophores, the obtained signal scales with ${N}_{f}=\tfrac{{k}_{{act}}}{{k}_{{act}}-{k}_{{bl}}}({e}^{-{k}_{{bl}}t}-{e}^{-{k}_{{act}}t})$, where *k*_act_ is the activation- and *k*_bl_ the bleaching-rate. The function was fitted to the experimental data to extract the parameters *k*_act_ and *k*_bl_ (Fig. 2d, lines shown with data). The extracted rates are plotted against the activation power in Fig. 2f. The activation rate scaled with the laser power as ${k}_{{act}} \sim {{P}_{{act}}}^{n}$, where $n=1.96\pm 0.16\,$, indicating a 2-photon process. The bleaching rate was determined to be 5-fold smaller and showed a fractional power dependency$\,{k}_{{bl}} \sim {{P}_{{act}}}^{1.54\pm 0.12}$, suggesting a mixture of linear and higher-order processes. Similar activation rate dependencies were observed following the same procedure for the two other dyes examined, HCage 620 ($n=2.95\pm 0.03$) and pPA-SiR ($n=2.48\pm 0.13$). Supplementary Fig. 3 contains the complete data. Evidently, all three dyes are activated by the 515 nm light.

### Two-photon photoactivation enables STED imaging in cells with improved contrast

Next, we examined the potential of the 2PA for sectioning in image acquisition and collected superresolution STED images, again of microtubules in chemically fixed cells. For this, we employed a 775 nm donut-shaped de-excitation beam (λ = 775 nm, τ ≈ 1.5 ns, f = 40 MHz, *P* = 40-70 mW) coaligned with the excitation focal spot. The fluorescence signal was detected roughly 1 ns after each excitation pulse to avoid contrast loss observed with long STED pulses (mitigated by so-called gated STED^28,29^).

**Fig. 3:**
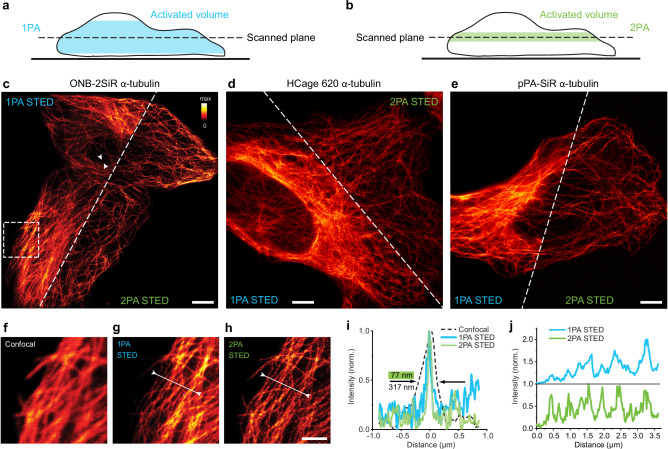
Two-photon photoactivation enables high-contrast STED imaging in cells due to improved optical sectioning. **a**, **b** Activated volume by scanning a sample plane (schematic) for 1PA **a** and 2PA **b**. **c**–**e** STED imaging of microtubules in U-2 OS cells labeled with ONB-2SiR **c**, HCage 620 **d** and pPA-SiR **e**, comparing prior photoactivation with 1PA at 375 nm and 2PA at 515 nm. An increased background is visible in the 1PA STED images, especially in thicker regions of the cells. Scale bars **c**–**e**: 5 µm. **f**–**h** Enlarged view of image region indicated in **c**, with confocal and 2PA STED image of the same region shown for comparison. Scale bar: 2 µm. **i** Intensity line profile in position indicated by arrows from both sides in **c**, for confocal, 1PA and 2PA STED, showing resolution improvement by STED. The resolution of the acquired image is similar for the 1PA and 2PA cases. **j** Intensity profile lines indicated by arrows and line in **g** and **h**, showing contrast improvement for 2PA. Not all microtubles are clearly visible in the profile line of the 1PA STED image, some are lost in the higher overall background. Source data are provided as a Source Data file.

To highlight the benefits of our chosen approach, we compared it to 1PA, reasoning that 2PA should provide a tighter axial confinement of the activated layer (Fig. 3b vs. Figure 3a), due to the rather sharply defined boundary of the 2PA volume. First, molecules were activated by 2PA during a scan of the FOV with the activation beam (λ = 515 nm, τ = 200 fs, *P* = 4 mW, t_dwell_ = 250 μs). Then, we collected the 2PA-STED image (Fig. 3c). In the following step, we activated the same sample region with 1PA (λ = 375 nm, cw, *P* = 220 µW, t_dwell_ = 250 μs) and collected a STED image once again. Side by side comparison images are shown in Fig. 3c. The activation efficiency in both activation modes (1PA and 2PA) allowed to record high-quality images (see enlarged views, including a confocal comparison, in Fig. 3f-h). We obtained super-resolved images of tubulin with measured full-width at half-maximum (FWHM) of individual filaments of ~75 nm (example in Fig. 3i), which corresponds to the diameter of the microtubule stained by primary and secondary antibodies determined by earlier studies (70-80 nm)^30^. For 1PA and 2PA, the measured widths of individual microtubules were similar, suggesting comparable resolution. The main advantage of 2PA results from the higher contrast which can be appreciated in the 2PA STED image and intensity profile comparisons (Fig. 3j). Some of the individual microtubules that are clearly visible in the 2PA STED image disappear in the background in the 1PA image. The higher contrast of the 2PA is especially noticeable in image regions where the sample is thicker, leading to a constant background in the 2PA case versus an increased background in response to labeled microtubules found above and below the focal plane for the 1PA case.

Similar STED images following 2PA were acquired with all three dyes, ONB-2SiR, HCage 620, pPA-SiR (Fig. 3c–e), demonstrating the higher contrast by 2PA. The results thereby also represent the first application of pPA-SiR for STED imaging. Since this dye is quenched over time due to a reaction with water, a more optimal imaging scheme collects the signal at every pixel right after activation. This can deliver up to 3-fold increases in detected fluorescence (with STED-beam active) compared to simple-frame activation (Supplementary Fig. 4).

### Sectioning in cells and tissue

**Fig. 4:**
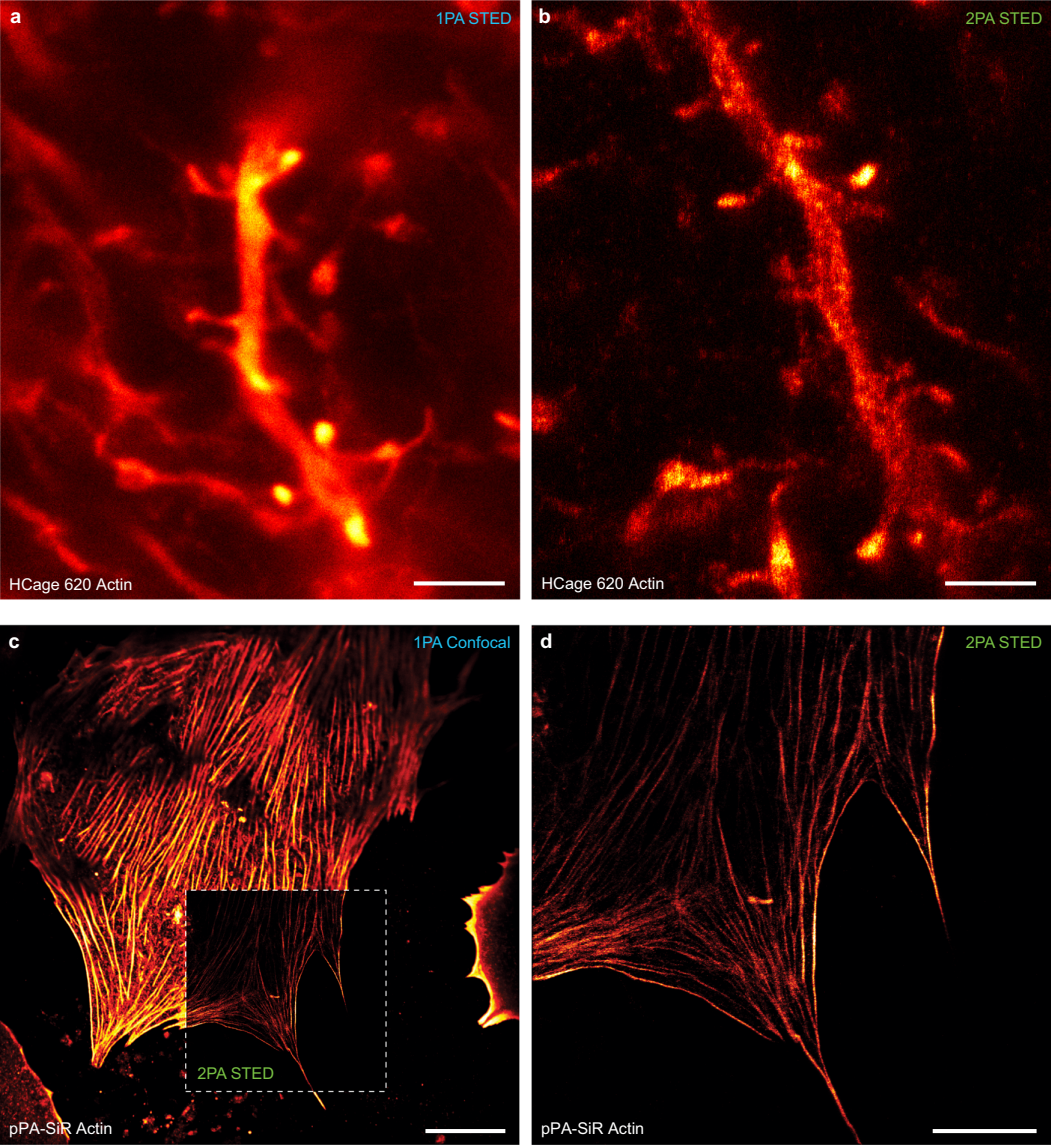
STED fluorescence nanoscopy with 515-nm two-photon photoactivation of silicon rhodamine dyes in cells and tissue. (**a,b**) Actin staining in mouse tissue sample (overexpressed LifeAct in layer V visual cortex dendrites) with HCage 620. (**a**) Image acquisition after 1PA exhibits significantly higher background due to out-of-focus fluorescence compared to the (**b**) STED image after 2PA. (**c,d**) Actin stained in hepatocytes grown in collagen with pPA-SiR as the fluorophore. (**c**) Confocal overview scan after 1PA. (**d**) STED image after 2PA. Scale bars: 2 µm (a,b), 20 µm (c), 10 µm (d).

Successful 2PA and subsequent STED imaging could be demonstrated in additional biological samples such as fixed primary hepatocytes and neurons (Fig. 4). To test the approach with a more challenging sample for STED microscopy, actin was labeled in the living animal by an injection of neuron-specific recombinant adeno-associated viral particles (AAVs) encoding Lifeact-EYFP^31^ to the layer V of visual cortex. Around 4 weeks after injection, the animal was sacrificed and the coronal slices prepared. To label actin in AAV-infected neurons we subsequently immunolabelled the fluorescent protein in the coronal slices by PA dyes (see Methods section). Both the actin in cortical layer V neurons within the paraformaldehyde-fixed brain slice, and actin imaged in primary hepatocytes grown in collagen exhibited similar increases in signal-to-background ratio in the 2PA case compared to classical 1PA. The advantage in image contrast for 2PA STED and its sectioning ability was readily appreciable when stepping through a volume of the brain tissue slice (Supplementary Movie 1).

**Fig. 5:**
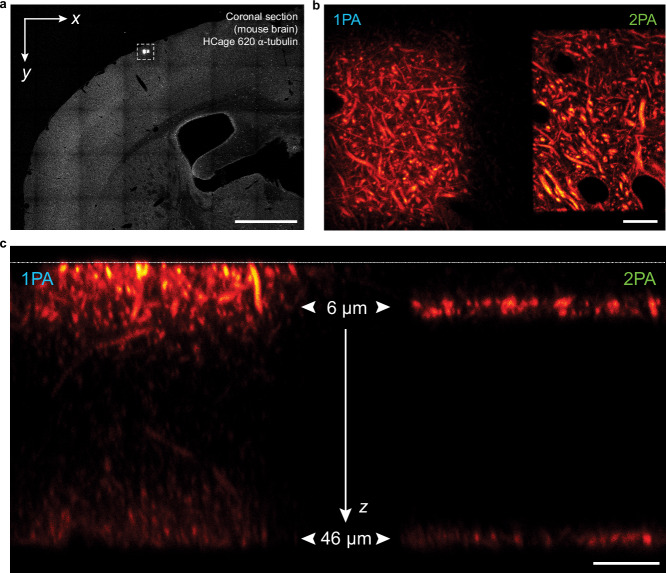
Two-photon photoactivation with 515 nm light provides selective access to optical sections in mouse brain tissue. To compare 1PA at 375 nm and 2PA at 515 nm, two layers at *z* = 6 µm and *z* = 46 µm were activated within two adjacent rectangular regions in a mouse brain cross section with HCage 620 labeling α-tubulin. **a** Confocal overview image after activation of two areas with 1PA (left) and 2PA (right). **b** Enlarged view of region shown boxed in (a), with the two activated regions. **c** A cross-sectional *y-z* scan shows the activated layers 6 µm and 46 µm inside the tissue slice (1PA on the left vs. 2PA on the right). The white dashed line at the top indicates the location of the coverglass. Two distinct layers are clearly visible in the 2PA case. Scale bars: 1 mm **a**, 10 µm **b**, **c**.

It is important to note that a similar contrast enhancement is achievable by total internal reflection (TIRF) excitation^32^ near the cover glass. The unique advantage of 2PA activation lies in the capability of optical sectioning in the depth of a sample, where TIRF is inapplicable. Therefore, we tested our approach performing 2PA and 1PA at the depth of 6 µm and 46 µm in a coronal section of a fixed mouse brain tissue in which tubulin had been labeled with HCage 620 (Fig. 5). In the case of 2PA, sharp borders of the signal after activation are apparent (Fig. 5a, b), and two individually activated layers are clearly distinguishable in a cross-sectional scan (Fig. 5c, right), confirming the multiphoton nature of the activation process. In contrast, 1PA shows activation along the optical path (Fig. 5c, left). The axially selected activation characterizing the 2PA process thus allows for significant reduction of out-of-focus fluorescence.

### Photoprotective effect of the sequential layer activation enables signal retention in 3D volumes with STED

**Fig. 6:**
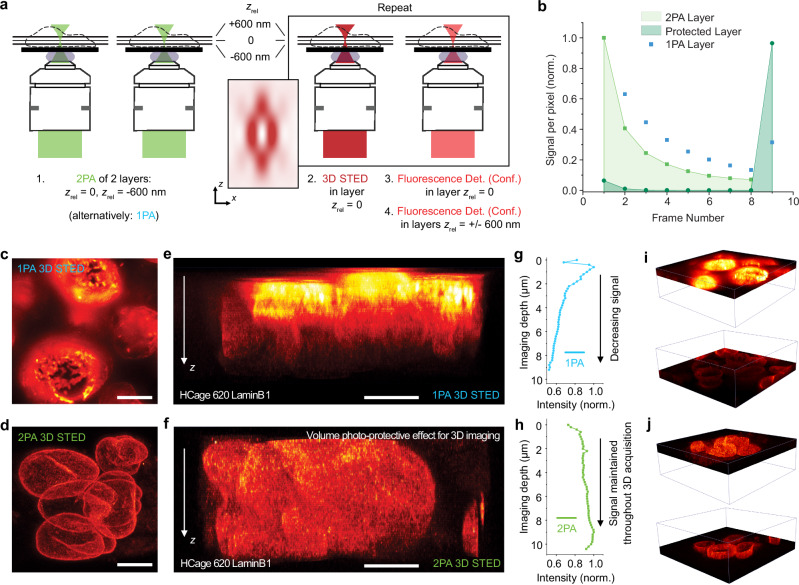
Photoprotective effect of the sequential layer activation enables high signal retention in STED imaging of 3D volumes. **a** Experimental scheme to test photoprotection of not activated layers. Det., Conf.: Detection, Confocal. **b** Fluorescence intensity of different layers of samples of microtubules in U-2 OS cells labeled with HCage 620 after repeated imaging scans with STED. The detectable signal of the 2PA layer (light green) is decreasing due to cumulative bleaching with the 3D donut. The not activated layer (dark green) is unaffected by the imaging scans, and full intensity can be recovered after all STED imaging scans. In the 1PA case (blue), the fluorescence cannot be recovered as that layer was activated at the start of the measurement due to the z-continuous, undisicriminating nature of linear activation. **c**–**j** Volume 3D STED imaging of LaminB1 stained in nuclei of neural stem cells. **c**, **d** Maximum intensity projections of image stacks along *z*, for 1PA **c** and 2PA **d**. **e**, **f** Cross-sectional y-z images for 1PA **e**, and 2PA **f**. **g**, **h** Average intensity (normalized to maximum signal) of the acquired layers vs. the corresponding imaging depth, up to ~10 µm, for 1PA **g** and 2PA **h**. An approximately even intensity distribution in the axial direction is achieved for selective activation of individual layers and subsequent STED imaging in the 2PA case, whereas the intensity of imaged layers continuously drops in the axial direction for 1PA due to the bleaching of already activated layers. **i**, **j** Comparisons of top, initially recorded vs. bottom, later recorded parts of the acquired 3D STED volume stack for 1PA **i** and 2PA **j**. Scale bars **c**–**f**: 5 µm. Source data are provided as a Source Data file.

An additional advantage to the increased contrast and control of the activation volume is the reduction of photobleaching. Fluorophores that are outside of the focal plane remain unactivated, as seen in the previous example, and are therefore not affected by the excitation and STED light during the image scan. This protection in the axial dimension is especially beneficial when imaging with a high-intensity 3D donut^14^. To estimate the magnitude of the protection effect with 2PA relative to 1PA, we performed experiments in fixed U-2 OS cells (Fig. 6a) with microtubules labeled with HCage 620. Two axial sample sections, designated center and below, were initially targeted by *xy*-scanning with the activation light with a distance of Δ*z* ≈ 600 nm. The central layer was then repeatedly imaged with STED (using the 3D donut), the fluorescent signal of the activated layer below was collected by confocal scanning after every STED imaging step and showed a drop in signal after every successive frame (Fig. 6b, light green). In the 2PA case, the layer with an equivalent distance from the central layer in the opposite direction above remained dark, and full fluorescence was recovered after all successive imaging scans by selective activation of that layer (Fig. 6b, dark green). In contrast, a full recovery of the fluorescence signal was not possible in experiments with 1PA, where the majority of fluorophores outside of the targeted layer were uncaged after the initial activation of the central layer and subsequently bleached during the successive imaging scans (Fig. 6b, blue). This effect is also visible in Fig. 6c–j, displaying nuclei in a neural stem cells colony where LaminB1 was immunostained with HCage 620 as the fluorophore. The 3D images were acquired in a 3D-STED configuration (relative power split in STED patterns: *P*_xy_ = 40 mW, *P*_z_ = 100 mW). They show a uniform signal distribution in all dimensions for the 2PA case (Fig. 6d, f, j), whereas the application of 1PA before 3D-STED resulted in a signal gradient over the process of imaging and corresponding imaging depth, due to successive bleaching (Fig. 6c, e, i). This is also apparent when quantifying the average detected fluorescence intensity per layer as a function of the imaging depth (Fig. 6g versus Fig. 6h).

### Sparse two-photon activation is compatible with nanometer-resolution MINSTED imaging

**Fig. 7:**
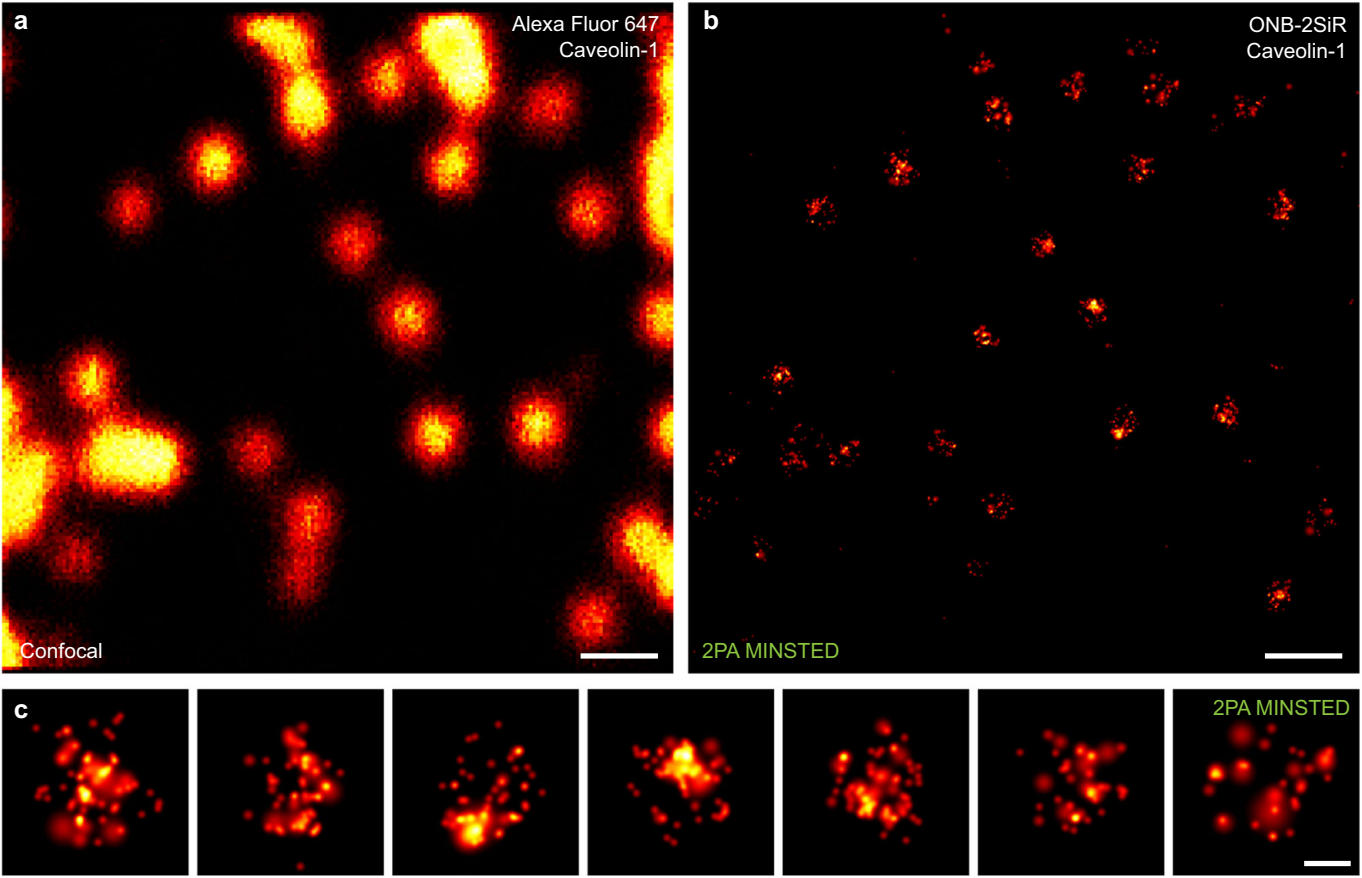
MINSTED fluorescence nanoscopy following two-photon activation of ONB-2SiR in samples of immunostained caveolin-1 in U-2 OS cells. **a** Confocal image (counter-stain with Alexa Fluor 647). **b** MINSTED reconstruction (median localization precision ~2 nm). **c** Enlarged views of caveolin-1 distributions. Scale bars: 300 nm **a**, **b**, 50 nm **c**.

When considering different nanoscopy concepts, it is clear that fluorophore PA is not necessarily required for the entire ensemble (in bulk), as in STED imaging. The coordinate-stochastic nanoscopy methods, for example, require single activated fluorophores spaced at sufficient distance to be individually localizable. Furthermore, novel concepts have emerged—notably MINFLUX^33–35^ and MINSTED^27^—which combine a photon-economic localization procedure through optical injection of a coordinate reference (by means of a light pattern with an intensity minimum) with the on-off switching at the level of single fluorophores. The derived imaging approaches have allowed to demonstrate fluorophore localization precisions and corresponding resolutions down to the single-digit nanometer range, as low as 1–3 nm. For this reason, we tested if it is possible to activate more sparsely, at the single-molecule level, via 2PA at 515 nm. Indeed, following activation at ~30 µW, MINSTED imaging resolved caveolin-1 in fixed U-2 OS cells (Fig. 7), providing images comparable in completeness to previous work^8^.

## Discussion

2PA of the three PA Si-R-based dyes in the visible range at 515 nm is possible with the use of a femtosecond laser source. The reported imaging experiments demonstrate that activation is robust and leads to images of complete structures, indicating that a substantial fraction of dyes remains intact and contributes fluorescence following the activation process.

The fact that two green photons can substitute for a single photon in the UV to provide the ~4.8 eV of energy required for activation arguably comes as a surprise to the field, which has traditionally associated two-photon sectioning in microscopy with near-infrared wavelengths^36,37^. Employing this wavelength, in the visible range, for activation is beneficial since it is much easier to deploy in a microscope compared to the regular UV wavelength used for 1PA. Many optical elements such as objective lenses exhibit very poor transmission of UV light and induce significant aberrations for light at the very short wavelengths. In contrast, the 515 nm light is well within the wavelength region of visible light for which most microscope objectives have been jointly optimized.

The 2PA shows clear benefits in confocal, 2D and 3D STED imaging modalities due to its additional contribution to optical sectioning, beyond the use of a pinhole and fluorescence inhibition of the STED-light pattern. A high degree of optical sectioning (~50–100 nm) can be achieved using TIRF illumination, but TIRF is restricted to regions very close to the coverslip and is not applicable deeper within samples. We can selectively activate individual layers inside of thick cortical tissue slices, with all dyes outside of the 2PA volume remaining unactivated. Those dyes do not compromise the imaging process due to additional signal, and they remain unbleached since they are not affected by the imaging process.

Single-molecule-level 2PA is also possible and can be combined with leading-edge single-molecule localization for ultimate localisation precision in fluorescence imaging. Overall, new opportunities arise as photoactivation moves to the visible regime and creates more flexibility in applying the new-found sectioning variant for various forms of fluorescence microscopy and nanoscopy.

## Methods

### Characterization setup and STED microscope

The STED measurements were taken on a custom-built confocal scanning microscope (Supplementary Fig. 2), which also served for the characterization of the PA fluorophores. A Leica 100x/1.4NA oil objective lens was used for cell imaging and the neuron imaging demonstration. All beams were scanned over the sample via two galvanometer mirrors in a 4f-relay arrangement, and passed a quarter-wave-plate to convert linear to circular polarization. The positioning of the sample was adjusted with a manual *x-y* stage, and focusing of the sample was facilitated by moving the objective with a *z*-piezo (PI-725-CDD).

The microscope setup was equipped with a STED laser (OneFive Katana, NKT Photonics, 640 ps pulse duration) operating at a wavelength of 775 nm and repetition rate of 40 MHz. The STED beam was expanded and subsequently split into two different paths with a polarizing beam splitter. In one path the formation of the *x-y* doughnut was achieved by a vortex phase plate (VPP-1a, RPC Photonics), and a custom-made 0-π phase plate was placed in the second path for the formation of the *z* doughnut. The beam was recombined with a second polarizing beam splitter. The ratio of the intensities between both beam paths was controlled with a half-wave plate. The fluorescence excitation beam at 640 nm (pulsed diode laser, PicoQuant LDH-D-C-640) was triggered to the STED laser. A femtosecond laser ( ~ 180 fs pulse duration, 20 MHz rate) at 515 nm (Halite, Fluence Technology) was used for 2PA of the dyes. The power in the focal plane was controlled via an AOTF (AA Opto Electronic). A two-prism pre-compressor system was used to correct for the pulse broadening introduced by the microscope optics. Conventional 1PA was achieved with a cw laser operating at 375 nm (Lambda Beam, RGB Lasersystems GmbH).

The detection features a PMT (Hamamatsu Photonics) for the characterisation of the PSF and an APD (SPCM-AQRH, Excelitas) for the confocal detection of the fluorescence signal. Gating of the detected signal can be achieved via custom software within LabVIEW by utilizing the Becker&Hickl SPC-150 time-correlated single photon-counting module.

### MINSTED microscope and imaging

MINSTED imaging was performed on the optical setup previously described^27^, with the addition of the 515-nm laser.

### Confocal microscopes

Big overview scans of tissue samples were recorded using a commercial confocal system, Leica SP8, with an excitation source at 633 nm and detection at 638 nm to 750 nm. Deep cross section images of cortical tissue were recorded on the previously published Quadscanner setup^38^ using an Olympus 60x/1.35NA silicon oil objective with an excitation laser at 640 nm and detection at 653–717 nm.

### Image data representation

A standard red-hot linear lookup table (LUT, as displayed in Fig. 3c) is used throughout the article to represent the image data.

### Sample mounting and imaging

Cell samples were mounted in PBS on an object slide and sealed with TWINSIL (picodent) or epoxy resin before placing them on the microscope. Acquisition of the STED images using line- or pixel-activation was achieved by controlling the individual laser sources and the detection with regards to the pixel clock generated by the custom FPGA software.

### Choice of activation power

An activation power of 4 mW was experimentally determined and then used, as it was a reasonable compromise between fast activation to yield high extractable signals (compare the faster-rising curve for 5 mW in Fig. 2d) and, at the same time, acceptable, low photobleaching. The experiments were aimed at obtaining individual STED image frames at high resolution, therefore the aim was to achieve single-shot activation of all fluorophores to maximize signal. A lower activation power would not have guaranteed that the majority of dyes will be found in the active state for STED image acquisition.

Using 2 mW of activation power would likely allow for more overall signal collection, but would require consecutive scans (to activate all fluorophores) and image stitching, thus prolonging image acquisition. The average 515 nm laser power needed for successful activation of pPA-SiR is notably lower ( < 1 mW) than that needed for ONB-2SiR and HCage 620 ( > 1 mW) (compare Supplementary Table 1).

Also, the optimal activation power varied with the sample age for some dyes, indicating an impact of the compound’s local chemistry on the photoactivation process. It was noticeable that the behavior of some dyes changed over time, starting from a few hours after the mounting of a sample. To achieve consistent fluorescence signals after activation, a significant reduction in the required 2PA power was needed. For time spans longer than 5 hours after mounting the sample, the required activation power was reduced significantly (from 4 mW to 2 mW) for the two dyes ONB-2SiR and HCage 620. Changing the imaging buffer or introducing oxygen scavenging systems had no effect. This behavior is possibly due to a partial thermal uncaging where the fluorophore loses one of the two caging groups without illumination of activation light. Care was therefore taken to image freshly mounted samples for those two dyes and ensure comparable conditions. No change of required activation power over time was detected for pPA-SiR.

### Pixel-based activation scheme

Pixel-based activation requires a more complex acquisition scheme and is more time-consuming compared to frame-based activation. However, for dyes that fade over time, this approach showed a significant improvement in contrast (Supplementary Fig. 4). To achieve the best performance, the acquisition scheme must be adjusted to the specific dyes being used. For dyes with high photostability, the improvement in contrast between pixel-based and frame-based activation was not as significant.

### Order of activation process

For pulse lengths of ~200 fs (515 nm), the activation rate of HCage 620 shows a dependency as a function of intensity very close to *n* = 3. To check whether this is in fact a three-photon activation process, we determined the activation rates with different pulse durations of the activation laser. For this purpose, pulses were stretched to different durations by introducing optical fibers (10 cm and 100 cm) into the beam path, and comparing data to ONB-2SiR, the dye which shows the clearest two-photon behavior based on the activation rate measurements (Table 1). The scheme for measuring the activation rates remained unchanged.

**Table 1:** Scaling of the activation rates with pulse duration

	k_act_ ( ~ 200 fs, no fiber stretcher)	k_act_ ( ~ 400 fs, 10 cm fiber)	k_act_ ( ~ 1200 fs, 100 cm fiber)
HCage 620	1.24 ± 0.16	0.62 ± 0.01	0.12 ± 0.01
ONB-2SiR	1.33 ± 0.08	0.72 ± 0.02	0.23 ± 0.02

Activation rates (in 1/frame) of HCage 620 and ONB-2SiR with fibers (pure silica) of different lengths placed in the beam path of the 515 nm activation laser.

Normalising the measured activation rates to the “no fiber stretcher” case (200 fs pulse duration), we obtain the relative scaling of the activation rates with pulse duration (Table 2).

**Table 2:** Relative scaling of the activation rates with pulse duration

Activation pulse duration at constant power (fs)	~200	~400	~1200
HCage 620	1	0.50	0.097
ONB-2SiR	1	0.54	0.173

Activation rates (in 1/frame) of HCage620 and ONB-2SiR scaling for different pulse duration (no fiber corresponds to 200 fs, 10 cm fiber to 400 fs, 100 cm fiber to 1200 fs).

A direct light-induced multiphoton process scales with intensity ~*I*^n^ and exhibits scaling with pulse duration *τ*^−n+1^. Equations for obtaining the scaling in pulse duration from the intensity scaling are provided in ref. ^18^. Therefore, please see Table 3 for theoretical scalings.

**Table 3:** Theoretical scaling with pulse duration

Theoretically expected scalings			
Ideal 1PA, *n* = 1	1	1	1
Ideal 2PA, *n* = 2	1	0.5	0.17
Ideal 3PA, *n* = 3	1	0.25	0.028

It is worth noting that, following activation, dyes like HCage 620 and pPA-SiR feature an equilibrium between the fluorescent product and a UV responsive transient dark state due to their chemical nature (in contrast to ONB-2SiR). The measured behavior for these dyes vs. pulse duration suggests that the process of activation is indeed, for the dominant part, also a 2-photon process as the signal scales with pulse duration approximately proportionally to τ^−1^. The scaling with close to *n* = 3 vs. intensity could be indicative of a more complicated photochemistry such as multistep absorption processes playing a role.

### Statistics and reproducibility

No statistics are derived. This is not a life science study with comparative analyses of a certain sample size. The proof-of-concept imaging experiments were each repeated at least 3 times with similar results.

### Animals

The described animal procedures were carried out in accordance with institutional regulations on animals use in research. Experiments performed on living animals were approved and authorized by the Lower Saxony State Office for Consumer Protection and Food Safety (Niedersächsisches Landesamt für Verbraucherschutz und Lebensmittelsicherheit (LAVES)). Sacrificing rodents for subsequent preparation of cultures did not require specific authorization or notification (Animal Welfare Law of the Federal Republic of Germany; Tierschutzgesetz der Bundesrepublik Deutschland (TierSchG)). All mice were housed with a 12 h light/dark cycle and *ad libitum* access to food and water.

### Cells

U-2 OS cells (ECACC, cat. 92022711, lot 17E015) were cultured in McCoy’s 5 A (modified) medium (Thermo Fisher, cat. 16600082) supplemented with 10% (v/v) FBS (Bio&Sell, cat. S0615), 1% (v/v) Sodium Pyruvate (Sigma, cat. S8636) and 1% (v/v) Penicillin-Streptomycin (Sigma, cat. P0781) in a humidified 5% CO_2_ incubator at 37 °C. Cells were seeded on coverslips 24 h before fixation.

The protocol of hepatocyte isolation and culture was modified from ref. ^39^. Briefly, an adult male mouse of C57BL/6 J background was sacrificed and immediately perfused via the hepatic portal vein with EGTA solution (pH 7.4) followed by collagenase solution. The perfused liver was transferred into a pre-warmed collagenase solution, mechanically dissociated and subsequently filtered through a 100 µm cell strainer. Cells were plated on coverslips that were pre-coated with collagen type I (Corning, cat. 354236) and cultivated in William’s Medium E (Gibco, cat. A12176-01) supplemented with 4% (v/v) Cell Maintenance Cocktail-B (Gibco, cat. A13448) and 10 µM Dexamethasone (Gibco, cat. A13449). Cultures were kept in a humidified 10 % CO_2_ incubator at 37 °C. The mice used for hepatocyte isolation were kept at 21 °C ambient average temperature and 50% humidity.

Neural stem/progenitor cells were cultured as previously described^40^. In brief, cortexes were dissected from C57BL/6 N mouse embryos of either sex at embryonic day E12.5, digested by 0.25% tripsin (Gibco, cat. 15090046) for 20 min at 37 °C and subsequently mechanically dissociated with a fire-polished, culture-medium-coated Pasteur pipette. Cells were plated on coverslips pre-coated with 0.2 % (w/v) gelatin in PBS and cultivated in KnockOut DMEM/F-12 medium (Gibco, cat. 12660012) supplemented with 2% (v/v) StemPro Neural Supplement (Gibco, cat. 10508-01), 1× GlutaMax (Gibco, cat. 35050061), 2 µg/µl Recombinant Human Basic Fibroblast Growth Factor (Gibco, cat. PHG0024), 2 µg/µl Recombinant Human Epidermal Growth Factor (Gibco, cat. PHG0314) and 0.5× Antibiotic-Antimycotic (Gibco, cat. 15240062) in a humidified 5% CO_2_ incubator at 37 °C. Mice used for the isolation of neural stem/progenitor cells were kept at 21 °C ambient average temperature and 50% humidity.

### Stereotaxic injections

The protocol of stereotaxic injection has been described previously in refs. ^38,41^. Briefly, an adult ( > 8 weeks old) mouse of C57BL/6 J background was anaesthetised by 1.0-2.0% isoflurane (Forene, Abbvie) in oxygen-enriched air (47.5 % oxygen, 50 % nitrogen, and 2.5 % carbon dioxide; Westfalen AG, Münster, Germany) and fixed into a stereotaxic apparatus (SG-4N, Narishige, Tokyo, Japan). The scalp was incised and a craniotomy was performed on the parietal bone above the visual cortex of the left hemisphere. A prepulled, tapered, borosilicate glass injection capillary (World Precision Instruments, Sarasota, FL, USA) was filled with a solution of pAAV-hSyn-Lifeact-EYFP virus^42^ diluted 1:5 in sterile artificial cerebrospinal fluid (ACSF; NaCl 126 mM, KCl 2.5 mM, CaCl2 2.5 mM, MgCl 1.3 mM, HEPES 27 mM, glucose 30 mM; pH 7.4). The capillary was subsequently lowered ca. 500 µm into the brain with an angle of 20° to the horizontal axis and the volume of 250–500 nl of virus-containing solution was injected using a pressure system (30 ms pulses delivered with 20 psi on manual command, TooheySpritzer, Toohey Company). The scalp was surgically closed by polyamide surgical suture (6.0/697H, Ethilon) immediately after retraction of the capillary and the animal was allowed to recover.

During the surgery perioperative analgesia was achieved by subcutaneous (s.c.) injection of Buprenovet (0.1 mg/kg mouse body weight, Bayer) and incision sites were analgised by s.c. injection of 2 % Lidocaine (Xylocaine, AstraZeneca). Through the whole surgery eyes were protected from dehydration by application of ointment (Bepanthen, Bayer) and a custom-built heating plate was used to maintain mouse body temperature. Analgesic and anti-inflammatory post-surgical care was achieved by s. c. administration of Carprofen (5 mg/kg, Zoetis) every 24 h for a minimal period of 2 days following surgery. Injected mice were kept at 20 °C ambient average temperature and 57% humidity.

### Intracardial perfusion fixation and slice preparation

Intracardial perfusion took place 3–4 weeks after stereotaxic viral injection. The mouse was anaesthetised by intraperitoneal injection of an overdose of Ketamine (Medistar)/ Xylasine (Dechra) and subsequently transcardially perfused with PBS, followed by 4 % (w/v) PFA in PBS. The brain was dissected and post-fixed by an overnight incubation in 4 % PFA/PBS at 4 ^o^C. Afterwards the fixative was removed, the brain was transferred into PBS and sliced into 60 µm thick consecutive coronal sections using a vibratome (VT1200S, Leica).

### Immunohistochemistry

Fixed slices were successively washed 2 times with Tris Buffer (TB), Tris Buffer Saline (TBS) and Tris Buffer Saline containing 0.5% (v/v) Triton-X 100 (TBST) (all with pH 7.6) for 15 min each at room temperature (RT). The slices were subsequently blocked with 10% (v/v) normal goat serum and 0.25% (w/v) bovine serum albumin (BSA) in TBST (blocking solution) for 1.5 h at RT. Sections were incubated for 72 h at 4 °C on a rocking plate with primary antibodies: (I) rabbit anti-GFP (Abcam, cat. 6556) diluted 1:300 or (II) rabbit anti-Tubulin (Abcam, cat. 18251) diluted 1:100 in blocking solution. Afterwards the slices were rinsed 4 times with TBST for 15 min each at RT and incubated with HCage 620^8^ conjugated goat anti-rabbit secondary antibody (Dianova, cat. 111-005-003) diluted 1:10 in TBST overnight at 4 °C on a rocking plate. Slices were successively washed 2 times with TBST, TBS and TB before being mounted with PBS.

### Immunocytochemistry for STED measurements

U-2 OS cells were fixed with ice-cold methanol for 5 min at -20 °C and subsequently blocked with 2% (w/v) BSA in PBS (blocking solution) for 10 min at RT. Cells were then incubated for 1 h at RT with primary rabbit anti-Tubulin antibody (Abcam, cat. 18251) diluted 1:100 in blocking solution. Afterwards the coverslips were washed with blocking solution for 10 min at RT and incubated for 1 h at RT with secondary anti-rabbit antibodies (Dianova, cat. 111-005-003) conjugated with: (I) ONB-2SIR^7,27^, (II) HCage 620^8^ or (III) pPA-SiR^6^ diluted in blocking solution. Cells were then washed 2 times with PBS and subsequently mounted in PBS.

Primary hepatocytes at the 15^th^ day of in vitro (DIV) culture were fixed with ice-cold methanol for 5 min at -20 °C and subsequently blocked with 10% (w/v) BSA in PBS at RT. Afterwards, cells were incubated with primary rabbit anti-actin antibody (Sigma, cat. A2668) diluted 1:100 in 2% (w/v) BSA in PBS. Cells were then successively washed with 0.5% (v/v) Triton-X in PBS and 2% BSA in PBS for 5 min each at RT and subsequently incubated with secondary anti-rabbit pPA-SiR^6^ conjugated antibody diluted 1:20 in 2% BSA in PBS for 1 h at RT. Coverslips were washed with PBS 2 times 15 min each at RT and subsequently mounted in PBS.

Neural stem/progenitor colonies at the 5^th^ DIV were fixed with ice-cold methanol for 10 min at -20 °C and subsequently blocked with 2% (w/v) BSA in PBS (blocking solution) for 10 min at RT. Cells were then permeabilized with 0.5% (v/v) Triton in PBS for 10 min at RT and incubated with primary rabbit anti-LaminB1 (Abcam, cat. 16048) antibody diluted 1:100 in blocking solution for 1 h at RT. Afterwards colonies were washed with blocking solution 2 times 10 min each and subsequently incubated for 1 h at RT with secondary anti-rabbit HCage 620^8^ conjugated antibody diluted 1:20 in blocking solution. Coverslips were then washed with PBS 2 times 15 min each at RT and subsequently mounted with PBS.

### Immunocytochemistry for MINSTED measurements

The caveolin-1 sample was prepared as described previously^8^, with an AlexaFluor647 counterstain serving to identify the plane of imaging. Briefly, U-2 OS cells were washed with PBS and subsequently fixed with 8% (w/v) PFA in PBS for 5 min at 37 °C. Afterwards, cells were permeabilized with 0.5% (v/v) Triton-X in PBS for 5 min at RT and subsequently blocked with 2% (w/v) BSA in PBS (blocking solution) for 10 min at RT. Cells were then incubated with primary rabbit anti-Caveolin-1 (Cell Signaling, cat. 3267) antibody diluted 1:200 in blocking solution for 1 h at RT. After that, cells were washed with blocking solution and incubated for 1 h at RT with secondary anti-rabbit antibodies conjugated with ONB-2SIR^7^ diluted in blocking solution. Cells were subsequently washed with blocking solution and counterstained for 1 h at RT with tertiary anti-goat antibody conjugated with Alexa 647 diluted 1:500 (Invitrogen, cat. A32849) in blocking solution. Samples were subsequently washed with PBS and incubated for 20 min with diluted silica shelled silver nanoplates (nanoComposix, SPSH1064-1M; 1 µg/ml in PBS). After washing the sample with PBS twice, they were mounted with PBS and closed using nail polish.

### Reporting summary

Further information on research design is available in the Nature Portfolio Reporting Summary linked to this article.

### Supplementary information

Supplementary Information

Reporting Summary

Description of Additional Supplementary Files

Supplementary Movie 1

### Source data

Source Data

## Data Availability

All data supporting the findings of this study are available within the paper and its Supplementary Information. The imaging data generated in this study have been deposited in the zenodo database under accession code 10.5281/zenodo.12731842. Source data are provided with this paper.

## Code availability

Images were rendered and evaluated using standard software tools (see Reporting Summary).
