# Supplementary material for: Bleaching protection and axial sectioning in fluorescence nanoscopy through two-photon activation at 515 nm
Source: Nat Commun. 2024 Aug 29;15:7472. doi: 10.1038/s41467-024-51160-9 (PMC11362616; doi:10.1038/s41467-024-51160-9)
Supplement: Supplementary file 1 — Supplementary Information [file 41467_2024_51160_MOESM1_ESM.pdf]

Supplementary Information

Bleaching protection and axial sectioning in fluorescence nanoscopy through two-photon activation at 515 nm

Jan-Erik Bredfeldt[1,2*], Joanna Oracz[1,*], Kamila A. Kiszka[1,*], Thea Moosmayer[1,2], Michael Weber[1], Steffen J. Sahl[1], Stefan W. Hell[1,2,†]

[1]Department of NanoBiophotonics, Max Planck Institute for Multidisciplinary Sciences, Göttingen, Germany
[2]Georg-August University School of Science (GAUSS), University of Göttingen, Göttingen, Germany
[3]Department of Optical Nanoscopy, Max Planck Institute for Medical Research, Heidelberg, Germany

*These authors contributed equally: J.-E.B., J.O., K.A.K.

†Correspondence should be addressed to S.W.H. (shell@mpinat.mpg.de).

Contents

Supplementary Fig. 1. Chromatic focal shift of high-quality objective lenses.

Supplementary Fig. 2. Optical setup.

Supplementary Fig. 3. Activation and bleaching rates vs. activation power for 2PA at 515 nm..

Supplementary Fig. 4. Pixel activation of pPA-SiR provides higher signal.

Supplementary Fig. 5. STED imaging with 1PA and 2PA: Actin staining in mouse tissue.

Supplementary Table 1. Parameters of photoactivation and image acquisition.

Supplementary References

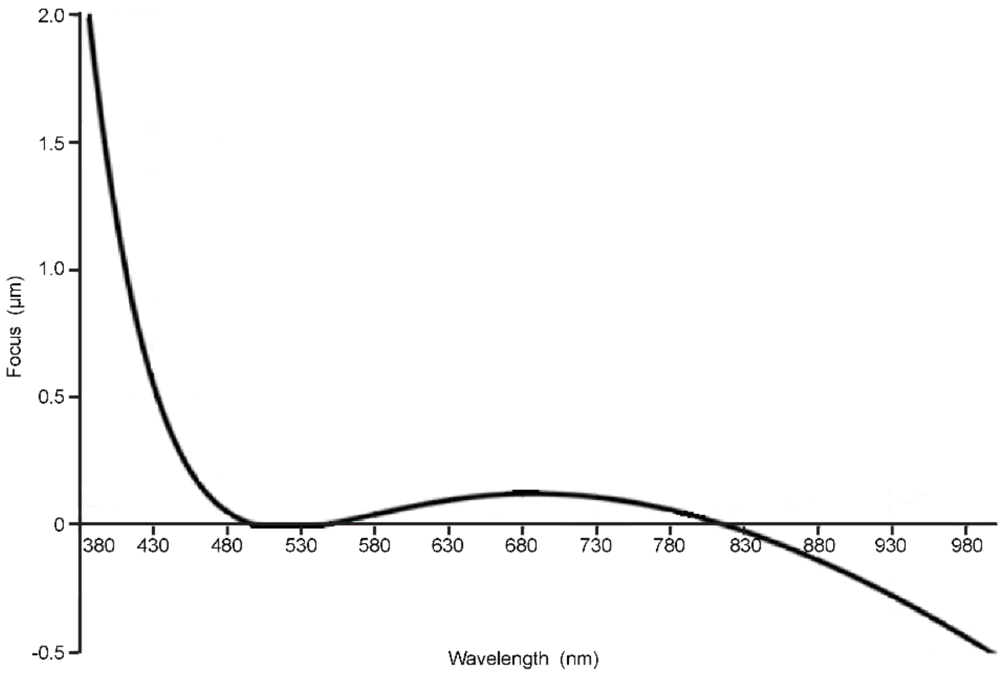

Supplementary Fig. 1. Chromatic focal shift of high-quality objective lenses. The longitudinal color error of even well-corrected objective lenses can be substantial outside the classical design range in the visible part of spectrum. Data for an HC PL APO 100x/1.40 OIL (Leica) objective is shown. In STED microscopy, all beams are typically being aligned to the optimal axial position of the STED beam (at e.g. 775 nm) as the doughnut zero determines the effective PSF (not the excitation beam). The offset to UV wavelengths for activation is therefore very substantial.

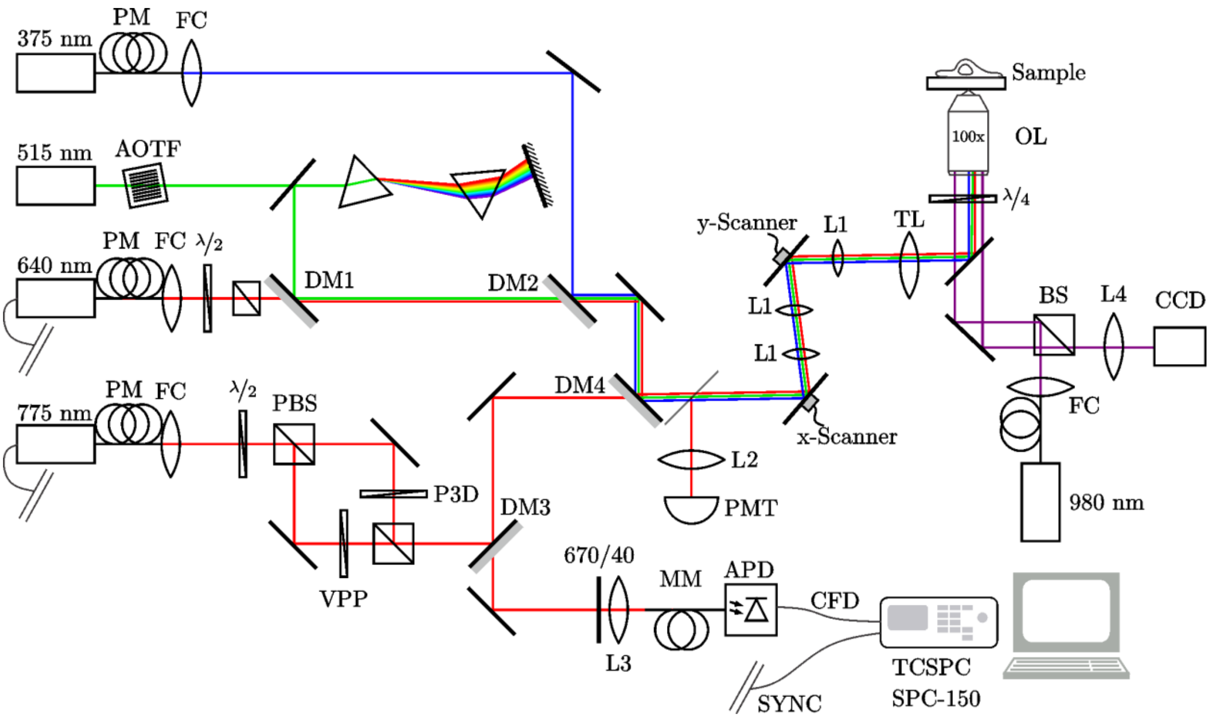

Supplementary Fig. 2. Optical setup. The microscope was equipped with four laser sources for imaging and activation. A 375 nm laser was used as reference excitation, representing classical 1PA of the dyes. The beam was spatially filtered through a PM fiber and coupled into the system via a dichroic mirror (415RDC, DM2). The 515-nm fs laser is used for 2PA. A two-prism pre-compressor was used to compensate for the pulse broadening induced by the optics in the microscope. The power of the activation laser was controlled with an AOTF. A 640-nm laser was used as an excitation laser source triggered to 40 MHz repetition rate of the 775 nm laser. The beam was filtered through a PM fiber and passed a half-wave plate and a Glan-Thompson prism for manual power control. The green beam was coupled to the red excitation beam with a dichroic mirror (532RDC, DM1). The 775-nm STED laser passed a half-wave plate and a polarizing beam splitter (PBS) to control the laser intensity in the two arms. One arm formed the *x-y* donut with the help of a vortex phase plate (VPP) whereas the other arm formed the 3D donut with the help of a custom made 0-π phase plate. Both beams were combined and coupled into the microscope with the dichroic mirrors 750SPRDC (DM3) and 635RDC (DM4). All beams passed over two galvanometric mirrors for *x-y* scanning, arranged in a 4*f* system with *f* = 40 mm triplet lenses (L1). The system was equipped with a *f* = 200 mm tube lens (TL). Before passing the objective lens (100x/1.4 NA Leica), the beams passed a quarter-wave plate. The detection featured a PMT for characterizing the PSF, and a removable pellicle was placed in the beam path before a *f* = 140 mm lens (L2). The fluorescence was detected with an APD placed after a focussing lens (*f* = 150 mm, L3) and two fluorescence filters (775-nm notch filter, 670/40). The sync signal from the 775-nm STED laser was the master trigger to the single photon counting module SPC-150 with APD counts as input (CFD). AOTF: acousto-optic tunable filter; APD: avalanche photodiode; BS: beam splitter; CCD: charge-coupled device; CFD: constant fraction discriminator; FC: fiber collimator; MM: multimode fiber; OL: objective lens; PBS: polarizing beamsplitter; PM: polarization-maintaining; PMT: photo-multiplier tube; TCSPC: time-correlated single-photon counting.

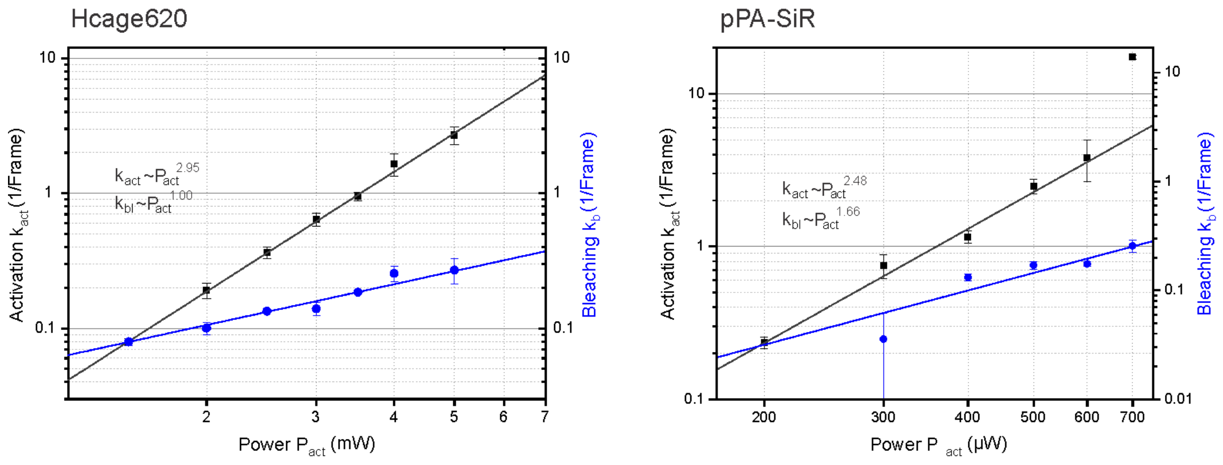

Supplementary Fig. 3. Activation and bleaching rates vs. activation power for 2PA at 515 nm. Activation and bleaching rates k_{act} , k_{bl} were extracted from fits to data from measurements according to the scheme from Fig. 2c with the dyes HCage 620 and pPA-SiR. Error bars represent standard error of the mean (s.e.m.). The data and fit results for ONB-2SiR are displayed in Fig. 2f. Source data are provided as a Source Data file.

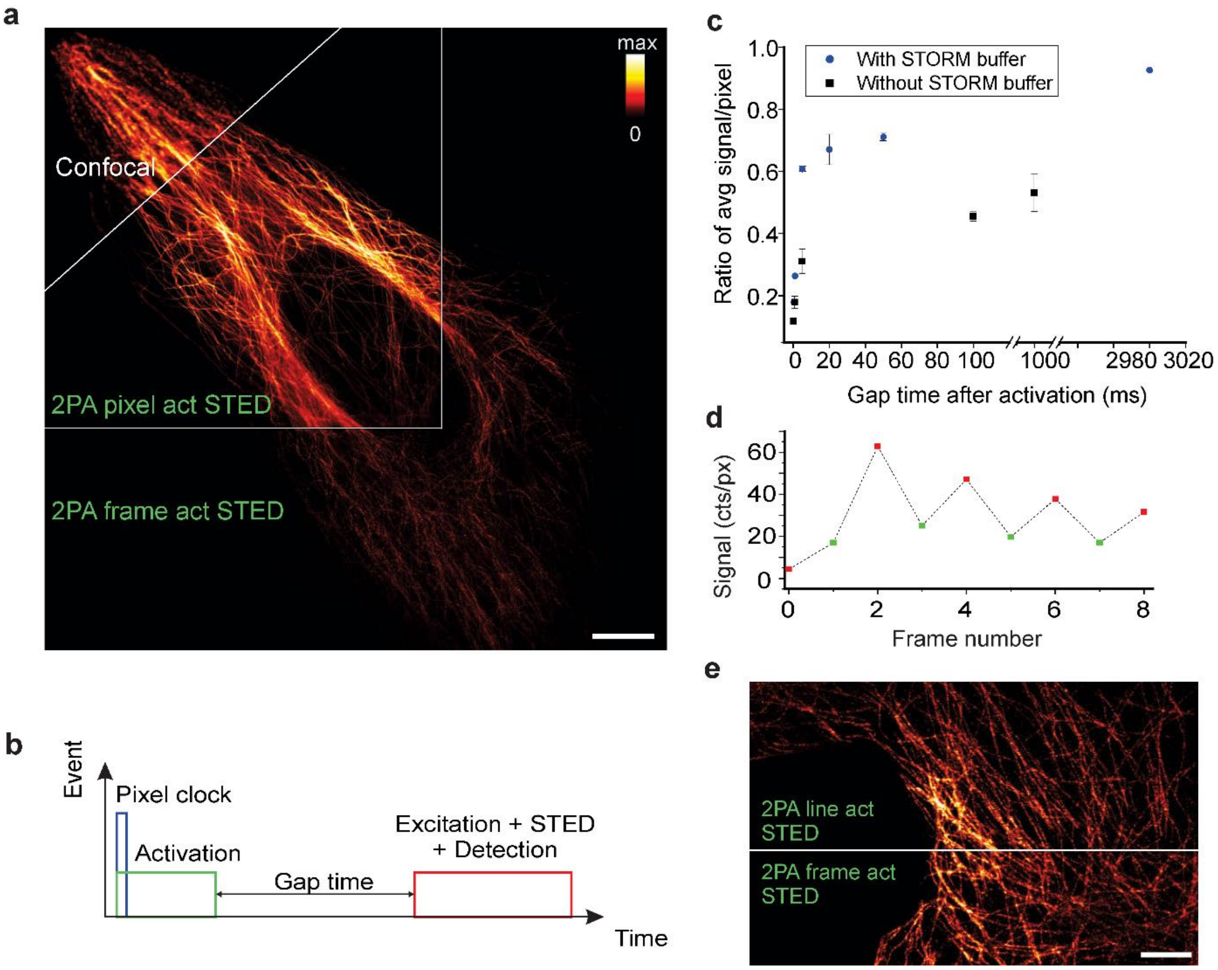

Supplementary Fig. 4. Pixel activation of pPA-SiR provides higher signal. (**a**) Pixel activation of pPA-SiR shows a 3-fold improvement of the obtained signal when imaging with STED compared to the simple frame activation. The activation time was 10 µs per pixel at 0.6 mW, STED 775 nm at 40 MHz at 90 mW. Scale bar: 10 µm. (**b**) For the pixel activation, the activation period with the 515-nm light was aligned to the pixel clock. The detection window together with the excitation and STED beams followed the activation window after a variable time gap until the next pixel clock. (**c**) Images acquired with pixel activation for the dye ONB-2SiR show a strong dependence of the detectable fluorescence on the time gap between the two windows. Very long gap times, on the order of ms or s, were needed for high signals. The gap time could be reduced when imaging with a buffer containing POC, glucose and BME (blue) compared to imaging in PBS buffer (black). The *y*-axis represents the obtained signal during the scan with activation and excitation vs. the signal obtained directly after the scan with excitation only. Error bars represent standard error of the mean (s.e.m.), which is similar to the marker size in some instances. This effect is also visible in (**d**), which shows the detected fluorescence during a pixel activation scan (green), and the scan following right after with excitation only (red) for four times, always on the same sample region. A similar behaviour was observed when imaging HCage 620. These observations may be explained by the equilibrium between fluorescent and non-fluorescent forms of the dyes (compare supplementary ref. 1). (**e**). The activation of single lines with the subsequent detection of the same line with STED (top) in order to mitigate the effect of long time gaps showed no significant improvement of the detectable signal for ONB-2SiR and HCage 620 when compared to regular frame activation (bottom). The example data shown is from an ONB-2SiR experiment. Scale bar: 5 µm. Source data are provided as a Source Data file.

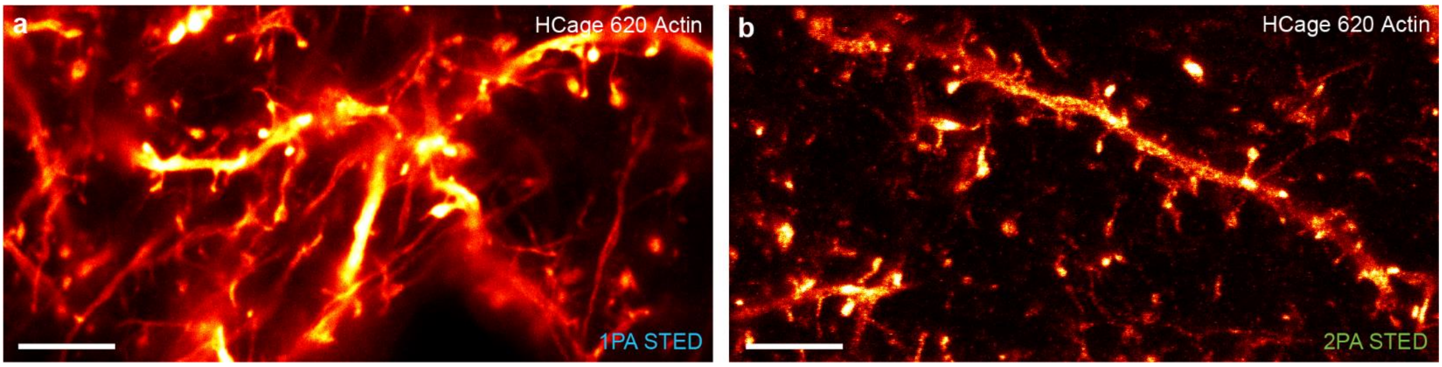

Supplementary Fig. 5. STED imaging with 1PA and 2PA: Actin staining in mouse tissue. Actin staining (HCage 620) in mouse tissue sample following 1PA (a) and 2PA (b). Scale bars: 5 µm (a,b).

Supplementary Table 1. Parameters of photoactivation and image acquisition (part 1).

Fig.	Measurement / Instrument	Objective	1PA avg. power (375 nm)	2PA avg. power (515 nm)	Pixel dwell time (activation)	Pixel size (activation)	Excitation avg. power (640 nm)	STED avg. power (775nm)	Pixel size	Pixel dwell time (imaging)	FOV size
2c, left	confocal, 1PA/2PA setup	Leica, HXC PL APO oil 100x/1.4NA	N/A	N/A	N/A	N/A	8 µW	N/A	195.3 nm x 195.3 nm	30 µs	50µm x 50µm
2c, right	confocal, 1PA/2PA setup	Leica, HXC PL APO oil 100x/1.4NA	N/A	4 mW	100 µs (2PA)	150 nm (xy)	8 µW	N/A	195.3 nm x 195.3 nm	30 µs	50µm x 50µm
3c	1PA STED,1PA/2PA setup	Leica, HXC PL APO oil 100x/1.4NA	220 µW	N/A	500 µs (1PA)	195 nm (1PA)	8 µW	40 mW (10 MHz, 1.5 ns gated)	24.4 nm x 24.4 nm	130 µs	50 µm x 50 µm
3c	2PA STED, 1PA/2PA setup	Leica, HXC PL APO oil 100x/1.4NA	N/A	4 mW	250 µs (2PA)	150 nm (2PA)	8 µW	40 mW (10 MHz, 1.5 ns gated)	24.4 nm x 24.4 nm	130 µs	50 µm x 50 µm
3d	1PA STED, 1PA/2PA setup	Leica, HXC PL APO oil 100x/1.4NA	130 µW	N/A	30 µs (1PA)	195 nm (1PA)	8 µW	100 mW (40 MHz, 1.5 ns gated)	20 nm x 20 nm	90 µs	50 µm x 50 µm
3d	2PA STED, 1PA/2PA setup	Leica, HXC PL APO oil 100x/1.4NA	N/A	~3 mW	30 µs (2PA)	150 nm (2PA)	8 µW	100 mW (40 MHz, 1.5 ns gated)	20 nm x 20 nm	90 µs	50 µm x 50 µm
3e	1PA STED, 1PA/2PA setup	Leica, HXC PL APO oil 100x/1.4NA	130 µW	N/A	30 µs (1PA)	195 nm (1PA)	8 µW	130 mW (40 MHz, 1.5 ns gated)	24.4 nm x 24.4 nm	120 µs	50 µm x 50 µm
3e	2PA STED, 1PA/2PA setup	Leica, HXC PL APO oil 100x/1.4NA	N/A	0.6 mW	10 µs (2PA)	24 nm (2PA)	8 µW	130 mW (40 MHz, 1.5 ns gated)	24.4 nm x 24.4 nm	140 µs	50 µm x 50 µm
3f	2PA STED, 1PA/2PA setup	Leica, HXC PL APO oil 100x/1.4NA	N/A	4 mW	250 µs (2PA)	150 nm (2PA)	8 µW	N/A	24.4 nm x 24.4 nm	30 µs	50 µm x 50 µm
4a	1PA STED, 1PA/2PA setup	Olympus, UPLSAPO60xS2 silicon oil NA 1.3 AA 0.3	130 µW	N/A	30µs (1PA)	195 nm (1PA)	8 µW	100 mW (40 MHz, 1.5 ns gated)	22.3 nm x 22.3 nm	130 µs	50 µm x 50 µm
4b	2PA STED, 1PA/2PA setup	Olympus, UPLSAPO60xS2 silicon oil NA 1.3 AA 0.3	N/A	3 mW	80 µs (2PA)	150 nm (2PA)	8 µW	80 mW (40 MHz, 1.5 ns gated)	22.3 nm x 22.3 nm	130 µs	50 µm x 50 µm
4c	1PA Confocal, 1PA/2PA setup	Leica, HXC PL APO oil 100x/1.4NA	13 µW	N/A	1 ms (1PA)	195 nm (1PA)	8 µW	N/A	63.5 nm x 63.5 nm	30 µs	130 µm x 130 µm
4d	2PA STED, 1PA/2PA setup	Leica, HXC PL APO oil 100x/1.4NA	N/A	0.6 mW	10 µs (2PA)	24 nm (2PA)	8 µW	90 mW (40 MHz, 1.5 ns gated)	24.4 nm x 24.4 nm	130 µs	50 µm x 50 µm

Fig.	Measurement / Instrument	Objective	1PA avg. power (375 nm)	2PA avg. power (515 nm)	Pixel dwell time (activation)	Pixel size (activation)	Excitation avg. power (640 nm)	STED avg. power (775nm)	Pixel size	Pixel dwell time (imaging)	FOV size
5a	confocal, SP8	Leica, HC PL APO CS2 oil 20x/0.75	130 µW	3.8 mW	300 µs (1PA and 2PA)	150 nm (1PA and 2PA)	/	N/A	0.76 µm	/	10 mm x 7.4 mm
5b	confocal, Quad Scanner (in vivo STED)	Olympus, UPLSAPO60xS2 silicon oil NA 1.3 AA 0.3	130 µW	3.8 mW	300 µs (1PA and 2PA)	150 nm (1PA and 2PA)	37 µW	N/A	50 nm (xy), 400 nm (z)	30µs	99.228 µm x 99.228 µm
5c	confocal, Quad Scanner (In vivo STED)	Olympus, UPLSAPO60xS2 silicon oil NA 1.3 AA 0.3	130 µW	3.8 mW	300 µs (1PA and 2PA)	150 nm (1PA and 2PA)	37 µW	N/A	50 nm (xy), 400 nm (z)	30µs	99.522 µm x 99.522 µm
6 c,e,g,i	1PA 3D STED, 1PA/2PA setup	Leica, HXC PL APO oil 100x/1.4NA	160 µW	N/A	30 µs (1PA)	20 nm (1PA)	8 µW	100 mW (40 MHz, 1.5 ns gated)	20 nm (xy), 200 nm (z)	130 µs	30 µm x 30 µm x 10.2 µm
6 d,f,h,j	2PA 3D STED, 1PA/2PA setup	Leica, HXC PL APO oil 100x/1.4NA	N/A	3 mW	30 µs (2PA)	20 nm (2PA)	8 µW	100 mW (40 MHz, 1.5 ns gated)	20 nm (xy), 200 nm (z)	130 µs	30 µm x 30 µm x 10.4 µm
7a	Confocal	Leica HC PL APO 100x/1.4 OIL CS2	N/A	N/A	N/A	N/A	5-10 µW	N/A	13 nm	20 µs	2.6 µm x 2.6 µm
7b,c	MINSTED	Leica HC PL APO 100x/1.4 OIL CS2	N/A	dynamically adapted	20 µs	50 nm (x) and 75 nm (y)	5-10 µW	50 mW (final)	N/A	N/A	2.6 µm x 2.6 µm
S4 a	2PA STED, 1PA/2PA setup	Leica, HXC PL APO oil 100x/1.4NA	N/A	0.6 mW	10 µs (pixel-by-pixel activation), 100 µs (frame activation)	24 nm (pixel-by-pixel activation), 195 nm (frame activation)	8 µW	90 mW (40 MHz, 1.5 ns gated)	31.7 nm x 31.7 nm	130 µs	65 µm x 65 µm
S5 a	1PA STED, 1PA/2PA setup	Olympus, UPLSAPO60xS2 silicon oil NA 1.3 AA 0.3	130 µW	N/A	30µs (1PA)	195 nm (1PA)	8 µW	100 mW (40 MHz, 1.5 ns gated)	22.3 nm x 22.3 nm	130 µs	50 µm x 50 µm
S5 b	2PA STED, 1PA/2PA setup	Olympus, UPLSAPO60xS2 silicon oil NA 1.3 AA 0.3	N/A	3 mW	80 µs (2PA)	150 nm (2PA)	8 µW	80 mW (40 MHz, 1.5 ns gated)	22.3 nm x 22.3 nm	130 µs	50 µm x 50 µm
Movie S1, left	1PA STED, 1PA/2PA setup	Leica, HXC PL APO oil 100x/1.4NA	205 µW	N/A	20µs (1PA)	195 nm (1PA)	8 µW	90 mW (40 MHz, 1.5 ns gated)	22.3 nm x 22.3 nm	130 µs	50 µm x 50 µm
Movie S1, right	2PA STED, 1PA/2PA setup	Leica, HXC PL APO oil 100x/1.4NA	N/A	3 mW	80 µs (2PA)	150 nm (2PA)	8 µW	80 mW (40 MHz, 1.5 ns gated)	22.3 nm x 22.3 nm	130 µs	50 µm x 50 µm

Supplementary Table 1. Parameters of photoactivation and image acquisition (part 2).

Supplementary References

1. Frei, M.S. et al. Photoactivation of silicon rhodamines via a light-induced protonation. *Nat. Commun.* **10**, 4580 (2019).
