# Supplementary material for: Bleaching protection and axial sectioning in fluorescence nanoscopy through two-photon activation at 515 nm
Source: Nat Commun. 2024 Aug 29;15:7472. doi: 10.1038/s41467-024-51160-9 (PMC11362616; doi:10.1038/s41467-024-51160-9)
Supplement: Supplementary file 3 — Description of Additional Supplementary Files [file 41467_2024_51160_MOESM3_ESM.pdf]

Supplementary Movie 1.
Two-photon activation STED in mouse brain slice. 1PA (left) vs. 2PA (right) STED recordings of actin in cortical layer V neurons within a paraformaldehyde-fixed brain slice (compare Fig. 4a,b and Supplementary Fig. 5). Nine steps of Δz = 200 nm were recorded, and this recorded sequence is displayed repeated 3 times within the movie.
